# Socioeconomic Deprivation Does Not Influence Disease Severity or Access to Dupilumab in Eosinophilic Esophagitis

**DOI:** 10.14740/gr2130

**Published:** 2026-04-27

**Authors:** Fatema Ali, Dong Xi

**Affiliations:** a Pediatric Gastroenterology, The University of Tennessee Health Science Center, Memphis, TN 38105, USA

**Keywords:** Eosinophilic esophagitis, Dupilumab, Area deprivation index

## Abstract

**Background:**

Studies have explored the relationship between eosinophilic esophagitis (EoE) and various factors such as race, gender, and geographic elements, including urbanization, population density, and climate zones. However, the relationship between socioeconomic advantage or disadvantage and key pathology findings—particularly the esophageal peak eosinophil count at diagnosis, as well as access to biologic therapy—has not been extensively studied in pediatric EoE. This study aimed to examine these relationships to uncover potential influences.

**Methods:**

This retrospective cohort study involved children aged 0 to 18 years evaluated in EoE clinic, including 16 patients who were refractory to other conventional therapies and ultimately treated with dupilumab. Details regarding eosinophil count per high power field (HPF) in the proximal and distal esophagus at the time of diagnosis were obtained from electronic medical records. Patients’ socioeconomic status was stratified using the Area Deprivation Index (ADI), which ranks neighborhoods based on socioeconomic disadvantage, calculated from the patients’ residential addresses. Data were analyzed using two-tailed *t*-test and multivariate regression analysis.

**Results:**

A total of 145 pediatric patients were included in the study. The patient population was spread relatively evenly across the different levels of the ADI. Our analysis showed no significant correlation between ADI and eosinophil count in the distal and proximal esophagus. One hundred twenty-nine patients demonstrated significant histological response to conventional non-dupilumab therapies. In a subset of 16 patients receiving dupilumab, the analysis revealed no significant difference of ADI, and baseline eosinophil count at the diagnosis in the proximal and distal eosinophil count compared with the overall cohort. However, dupilumab treatment was associated with a significant reduction in eosinophil counts, suggesting improved histologic outcomes compared with non-dupilumab therapies.

**Conclusions:**

This study examined the potential impact of socioeconomic disparities on pediatric patients with EoE, including those who were refractory to other previous therapies and ultimately treated with dupilumab. Our results suggest that socioeconomic deprivation, as measured by the ADI, is not associated with baseline histologic severity in pediatric EoE. Dupilumab demonstrated significant therapeutic benefit in patients who were refractory to other therapies, and access to dupilumab was not influenced by ADI.

## Introduction

Eosinophilic esophagitis (EoE) is a chronic immune/antigen-mediated disease characterized clinically by symptoms related to esophageal dysfunction and histologically by eosinophil-predominant inflammation [1]. Presentation in pediatrics varies between feeding intolerance, vomiting and abdominal pain at a younger age, to dysphagia and food impaction in adolescence [2]. A peak eosinophil count ≥ 15 intraepithelial eosinophils in at least one high power field (HPF) in an esophageal biopsy remains the gold standard for the pathologic diagnosis of EoE [1, 3, 4]. It has been incorporated with other histopathologic features in tools used to assess EoE severity [5–7].

EoE prevalence is known to be influenced by race and sex [8]. In addition, studies have shown that EoE prevalence varies by geographic region [9, 10], degree of urbanization [11], and climate [12]. The relationship between socioeconomic disparities and EoE pathology at diagnosis in children remains poorly studied. Social disparities among children with EoE, were recently studied [13] in a large retrospective study conducted in Colorado, United States. The study reported mixed findings regarding the association between the level of socioeconomic deprivation and specific aspects of EoE diagnosis and care, including age at diagnosis, age at esophageal dilation, age at seeing a feeding therapist or dietitian, and esophageal food foreign body removal. In adults with EoE, socioeconomic status was not associated with the risk of esophageal food impaction in a recent study [14]. Furthermore, while racial and ethnic disparities have been reported to possibly impact access to dupilumab among children receiving it for atopic dermatitis [15], socioeconomic disparities in dupilumab accessibility have not yet been studied in patients with EoE.

The Area Deprivation Index (ADI) has been widely used as a relatively accurate proxy measure of neighborhood-level socioeconomic deprivation. ADI is a validated, neighborhood-level measure of socioeconomic disadvantage derived from US Census data. It incorporates 17 indicators related to income, education, employment, and housing quality, including measures such as poverty, educational attainment, unemployment, and housing crowding [16–19]. These variables are combined into a composite score that reflects the relative level of socioeconomic deprivation of a geographic area, typically at the census block group level. ADI scores are standardized nationally and can be expressed as either a continuous measure or as percentiles ranked from 1 to 10, with higher values indicating greater neighborhood-level deprivation.

This study aims, first, to examine the correlation between socioeconomic deprivation and esophageal eosinophil counts in pediatric patients with EoE; second, to assess the clinical benefits of dupilumab in refractory EoE patients; and third, to evaluate the influence of socioeconomic disparities on dupilumab accessibility.

## Materials and Methods

### Ethics statement

This study was approved by the Institutional Review Board of the University of Tennessee Health Science Center and Le Bonheur Children’s Hospital. This study was conducted in compliance with the ethical standards on human subjects as well as with the Helsinki Declaration.

### Methodology

This retrospective cohort study analyzed children (ages 0 to 18) diagnosed with EoE using the Updated International Consensus Diagnostic Criteria for Eosinophilic Esophagitis [20]. The study included patients seen at the EoE Multidisciplinary Clinic at Le Bonheur Children’s Hospital (Memphis, Tennessee), which serves a regional catchment area including middle and western Tennessee (TN), northeastern Arkansas (AR), and northern Mississippi (MS). Based on institutional practices, patients with newly diagnosed EoE, those with allergic comorbidities, and those requiring escalation of treatment are referred to the EoE Multidisciplinary Clinic. As a result, this cohort captured the entire population of pediatric EoE patients managed at our institution, including all those initiated on dupilumab therapy. These patients were seen in the EoE clinic either at initial presentation, after referral from the general gastroenterology clinic, or following identification during hospitalization for acute presentations such as food impaction. Patients with other diagnoses were excluded from the study, including those with moderate to severe neurodevelopmental disorders or global developmental delay that impair adherence to study procedures, enteral feeding dependence, or the presence of other gastrointestinal or systemic conditions that could confound histologic findings, such as inflammatory bowel disease, celiac disease, or immunodeficiency.

The study patients were seen in the EoE Multidisciplinary Clinic between 2022 and 2024, for either an initial consultation or a follow-up visit. This time frame was selected to align with the period around the Food and Drug Administration (FDA) approval of dupilumab for pediatric EoE [21, 22], allowing for the evaluation of its access during the early implementation period. Patients were considered to have treatment-refractory EoE if they demonstrated persistent symptoms of esophageal dysfunction and ongoing histologic activity (≥ 15 eosinophils/HPF) despite adequate trials of conventional therapies (dietary elimination, proton pump inhibitor (PPI), and swallowed topical corticosteroid (STC)). Escalation to dupilumab therapy was considered for patients with refractory disease, intolerance to or contraindications for conventional therapies. For all patients, clinical and pathological records were reviewed retrospectively from the time of EoE diagnosis through their visits during the study period. Details regarding demographics, and eosinophil counts in the proximal and distal esophagus at the time of diagnosis and after the treatments, as well as details on the dupilumab treatment course in patients who received it, were extracted from electronic medical records.

Patients’ socioeconomic status was stratified using the ADI, calculated from the patients’ residential addresses by using the following formula ADI = ∑ (factor-derived weight × census variable value extracted from socioeconomic domains [16]. The ADI, which has been linked to health outcomes, is a multidimensional tool that measures socioeconomic disadvantage at the neighborhood level in the United States based on US Census data and American Community Survey data [16–19]. The ADI ranks census blocks, the smallest geographic area for which the Bureau of the Census collects and tabulates decennial census data from 1 to 100 at a national level or 1 to 10 at a state level, with the higher scores indicating more socioeconomic deprivation. State-level ADI was used in the study.

### Statistics

The correlations between ADI and baseline eosinophil count were analyzed and compared between the overall EoE cohort and the subset of patients who received dupilumab. Data were analyzed and presented using descriptive analysis, *t*-test, correlation coefficient analysis, and multivariate linear regression analysis. A P value of < 0.05 was used as statistically significant.

## Results

### Patients’ demographics and ADI distribution

Using the International Classification of Diseases, 10th Revision (ICD-10) codes for eosinophilic esophagitis across electronic medical record system and our local EoE patient database, we identified 208 patients with EoE as the study denominator, of whom 145 pediatric patients were included in the study. Demographics are described in Table 1. Most patients were White males (68% males, 32% females). Age ranged from 0 to 18 years, with the mean age at diagnosis being 8.6 ± 5.05 years.

**Table 1:** Patient Demographics and ADI Distribution

Age at diagnosis (1–18 years), mean ± SD	8.6 ± 5.05
Sex	
Male	68%
Female	32%
Race	
White	73%
Black	22%
Hispanic	3%
ADI distribution	
1–3	42%
4–7	26%
8–10	32%

ADI: Area Deprivation Index; SD: standard deviation.

### No correlation between ADI and baseline disease severity

**Figure 1:**
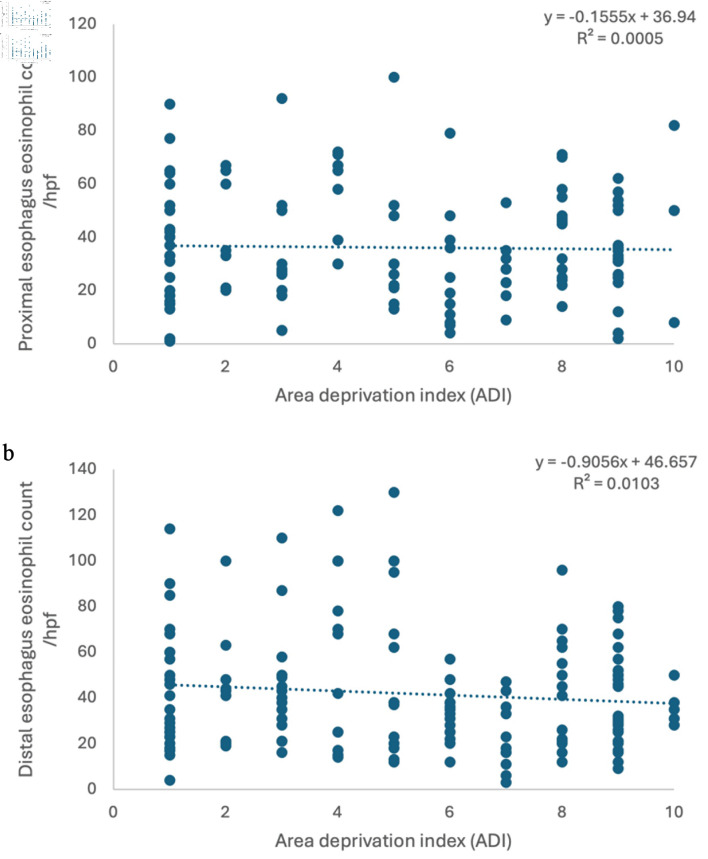
No correlation was found between ADI and eosinophil count in proximal esophagus (a) and distal esophagus (b). ADI: Area Deprivation Index.

The study population included patients from regions of three contiguous states (TN, MS, and AR) and was relatively evenly distributed across ADI levels, with 42% classified as low deprivation (ADI 1–3), 26% as middle deprivation (ADI 4–7), and 32% as high deprivation (ADI 8–10) (Fig. 1). The median ADI was 5 (Q25–Q75: 3–8). Our analysis showed that there is no significant correlation between ADI and baseline eosinophil count in the proximal esophagus at diagnosis (R^2^ = 0.0005, correlation (r) = 0.02, P value = 0.804), nor between ADI and eosinophil count in the distal esophagus at diagnosis (R^2^ = 0.0103, correlation (r) = –0.1, P value = 0.214) (Fig. 1a, b).

### Dupilumab demonstrates significant histological improvement in refractory EoE

**Figure 2:**
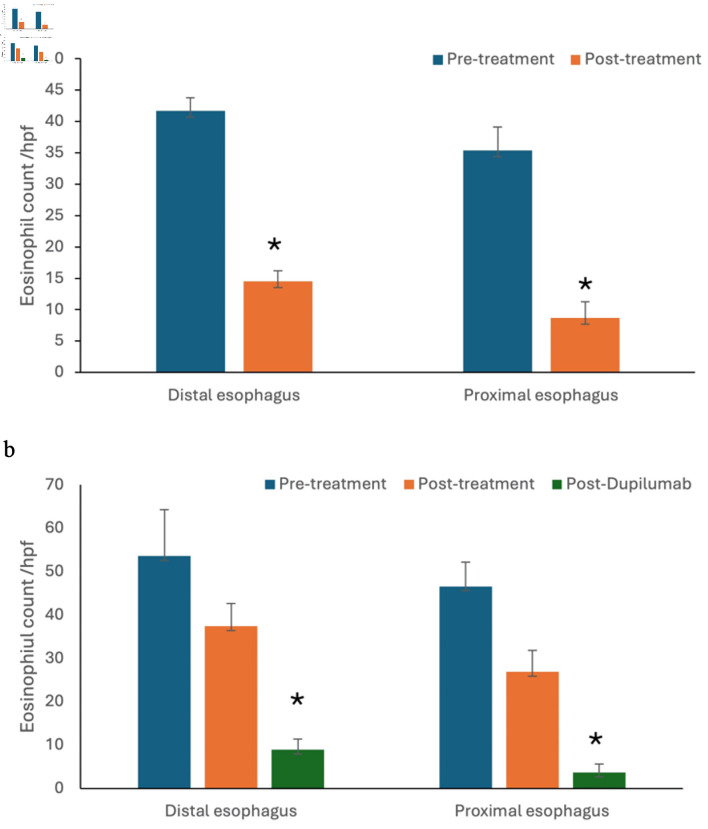
In addition to patients who responded to non-dupilumab therapies (a), a subset of refractory patients (b) demonstrated significant histological improvement with dupilumab (*P value < 0.05).

One hundred twenty-nine of the total 145 patients, described in Table 2, demonstrated significant histological response to non-dupilumab therapies including dietary elimination, PPI, and/or STC, with reduction of both proximal and distal esophageal eosinophil count (P value < 0.05) (Fig. 2a). No significant clinical and histological response was observed in other 16 patients. These 16 patients, described in Table 3, of the total of 145 patients, were subsequently treated with dupilumab. Follow-up endoscopy was performed on these patients 6 months after initiation of dupilumab and demonstrated significant histological improvement (distal esophagus eosinophil count/HPF: 37.4 ± 5.26 versus 8.92 ± 2.49; proximal esophagus eosinophil count/HPF: 26.9 ± 4.95 versus 3.67 ± 2.01; P value < 0.05) (Fig. 2b)

**Table 2:** Demographic Characteristics of Patients Who Responded to Conventional Therapies

Age at diagnosis (1–18 years), mean ± SD	8.56 ± 5.13
Sex, n (%)	
Male	89 (69%)
Female	40 (31%)
Race	
White	74%
Black	22%
Hispanic	2%
Baseline eosinophil count/HPF	
Distal esophagus	41.7 ± 2.14
Proximal esophagus	35.4 ± 3.79
Specific treatment received, n (%)	
Dietary elimination	2 (2%)
PPI	38 (29%)
STC	59 (46%)
PPI + STC	30 (23%)

SD: standard deviation; HPF: high power field; PPI: proton pump inhibitor; STC: swallowed topical corticosteroid.

**Table 3:** Demographic Characteristics of Patients Who Responded to Dupilumab

Age at diagnosis (1–17 years), mean ± SD	8.91 ± 4.47
Sex, n (%)	
Male	10 (63%)
Female	6 (37%)
Race	
White	69%
Black	25%
Hispanic	6%
Comorbidities	
Atopic condition(s)	75%
None	25%
Symptom severity	
Food impaction	13%
Prior esophageal dilation	6%
Treatment(s) prior to dupilumab	
PPI and STC	82%
STC only	18%
Number of failed treatment(s)	
Two treatments	100%
Duration of previous treatment(s)	3–12 months
Reason for discontinuation	
Inadequate response	62%
Intolerance	25%
Non-compliance	13%

SD: standard deviation; PPI: proton pump inhibitor; STC: swallowed topical corticosteroid.

### No influence of ADI on dupilumab accessibility

**Figure 3:**
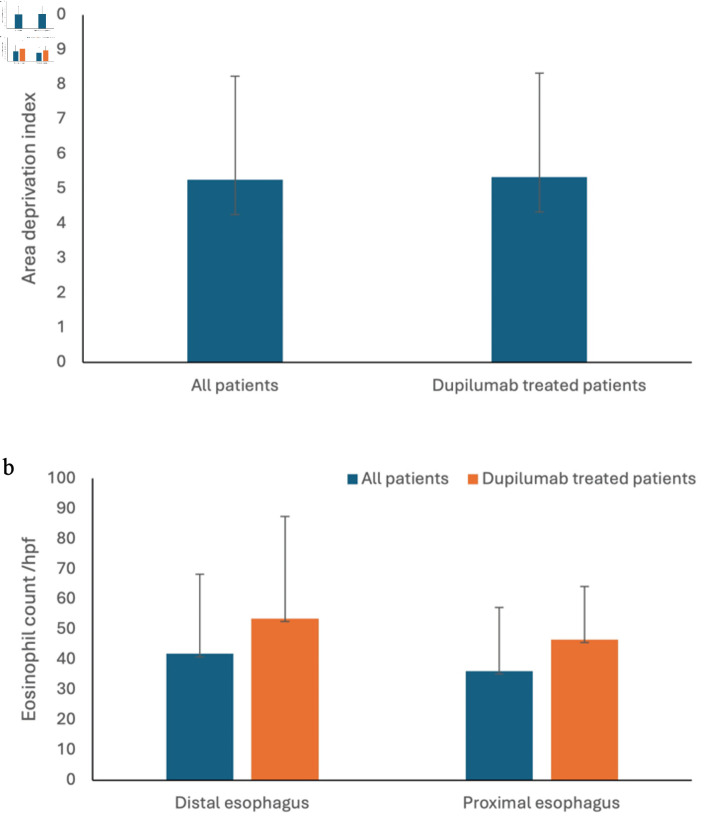
No difference was found in ADI (a) and eosinophil count in distal and proximal esophagus (b) between the overall cohort and dupilumab-treated patients. ADI: Area Deprivation Index.

The primary indication for switching to dupilumab was uncontrolled EoE despite maximum therapy, and/or intolerance, non-compliance to conventional therapies. All 16 patients achieved clinical remission after starting dupilumab. No significant difference of ADI was found between the overall cohort (mean ± standard deviation (SD): 5.25 ± 2.97) and dupilumab-treated patients (mean ± SD: 5.33 ± 2.99) (P value > 0.05) (Fig. 3a). Similarly, no significant differences in eosinophil counts were observed between the overall cohort (36.1 ± 21.2) and dupilumab-treated patients (46.6 ± 17.7) in the proximal esophagus (P value > 0.05), nor in the distal esophagus (P value > 0.05) (overall cohort: 41.9 ± 26.4; dupilumab-treated patients: 53.6 ± 33.9) (Fig. 3b).

## Discussion

Our analysis of 145 eligible pediatric EoE patients from a catchment area spanning parts of three neighboring states was consistent with prior studies, demonstrating a demographic predominance of White males [8, 13, 23]. We found no significant correlation between ADI and eosinophil count in the proximal and distal esophagus in the overall cohort. A similar pattern was reported by Mehta et al in their large retrospective study examining ADI in children with EoE [13]. Our analysis was retrospective and correlational in nature, additional unmeasured confounding factors within the EoE Multidisciplinary Clinic, including earlier referral and evaluation of infants and toddlers with atopic comorbidities such as eczema, food allergy, or asthma, and facilitated access to specialty care through public insurance may have influenced the observed results.

Among the 16 patients who were refractory to other therapies and ultimately received dupilumab, ADI distribution was evenly spread across deprivation levels and was comparable to the overall ADI distribution in the EoE cohort, suggesting that higher socioeconomic deprivation did not significantly impact access to dupilumab in this population. It is worth noting that all three states represented in our cohort—TN, MS, and AR—have higher-than-average Medicaid (public insurance) enrollment rates [24–26] compared to the national average of approximately 21% [27]. This broader reliance on public insurance may have contributed to more equitable access to dupilumab therapy.

All the 16 patients who were started on dupilumab achieved clinical and histological remission, suggesting a favorable response to treatment in this subgroup of patients. Research has already shown potential racial and ethnic disparities in dupilumab access for patients with atopic dermatitis [15]. Our findings showed that different levels of ADI have no significant influence on dupilumab accessibility for the patients who were initially started on this therapy. However, when layered with socioeconomic challenges, maintaining long-term dupilumab access may be even more difficult. This observation highlights the need for further investigation in a longer treatment duration and in a larger pediatric EoE population, as the cost-effectiveness of dupilumab, while suggested in other conditions like atopic dermatitis [28], has not yet been fully established for EoE [29].

This study has its limitations. It was conducted at a single center with a relatively small sample size, which may limit generalizability. While this cohort captured a population of pediatric EoE cases managed at our institution, several factors may introduce selection bias. The EoE Multidisciplinary Clinic mostly sees patients with complex diseases or those with allergic comorbidities. Notably, all patients treated with dupilumab during the study period were included, while the study cohort was limited to patients seen between 2022 and 2024, aligning with the FDA approval period of dupilumab for pediatric EoE. This may have excluded individuals diagnosed prior to this period who did not return for follow-up, such as those with well-controlled disease.

The small cohort of patients on dupilumab (n = 16) further limits the generalizability of our findings. Additionally, a longer follow-up period would have provided better insight into treatment sustainability and cost-effectiveness of dupilumab therapy.

While we used a standardized index ADI to assess social deprivation, our cohort included patients from three different states (TN, MS, and AR). All three states have higher levels of poverty than the national average [30–32]. Although ADI includes poverty rate as one of its components [19], the same ADI score may not reflect equivalent deprivation across states. Furthermore, while ADI is a useful proxy for social determinants of health, it may create a bias and does not capture individual-level socioeconomic data, which may more directly influence access to care.

### Conclusions

In summary, this study highlights that socioeconomic deprivation, as measured by ADI, is not significantly correlated with the disease severity in pediatric EoE, the accessibility to dupilumab and therapeutic response for the patients who are refractory to conventional therapies. Due to the limitations such as small sample size, the use of neighborhood-level measure, unsured individual insurance and economic status, and the lack of multivariate adjustment, we acknowledge that this study is underpowered to exclude modest but clinically meaningful disparities. This work may serve as a foundation for future research, ideally expanded to a multicenter level with longitudinal follow-up, to better understand the correlation between socioeconomic deprivation and the sustained accessibility for pediatric population, as well as long-term clinical outcome.

## Data Availability

The data supporting the findings of this study are available from the corresponding author upon reasonable request.

## Abbreviations

EoE: eosinophilic esophagitis
HPF: high power field
ADI: Area Deprivation Index
TN: Tennessee
AR: Arkansas
MS: Mississippi
